# Writers' Brains

**Published:** 1892-01-16

**Authors:** 


### The Doctor's Armchair.

### WRITERS' BRAINS.

Every writer in Europe feels an interest which he never felt before in the private lunatic asylum where M. Guy de Maupassant is now confined. So far as can be gathered from published statements, M. de Maupassant owes his present calamity to three circumstances: he worked at high pressure: he took stimulants to whip up his brain beyond its natural working capacity: and then he took sedatives to quiet the brain down when he wished to go to sleep. Writers have to a large extent the making of the modern world in their hands. They have taken the place of ancient prophets, modern preachers, and despotic rulers. They speak to the world in the only tones which can really command its attention—the tones of argument, of persuasion, of knowledge. Those teachers teach the world; but who teaches the teachers?

Whatever be the process by which the modern writer produces his light, it is of the first importance that the light be pure and strong, in order that those who come to it may see all things exactly as they are. The writer borrows light from manifold sources, but he himself is a burning luminary of more or less intensity and brightness. He, the borrower, borrows according to his own nature, and the light borrowed mingles and combines with the light that is in himself. The radiance he sheds upon the world is not merely a reflected radiance, but a light which is made up of his own personal luminosity combined with that which he has borrowed from other sources. The personal element is always the most important factor to be considered in a writer's works, and in the analysis of the personal element the most important factor is ever that of sanity. A writer must be first, and above all things, sane. There are degrees of sanity. Sanity in the highest degree is the endowment of comparatively few writers, even of the first rank. Shakespeare was eminently sane; Carlyle was often conspicuously wanting in sanity. Moses was of sound and wholesome mind; the prophet Elijah sometimes abandoned the seat of judgment, and enacted the rôle of the religious fanatic.

Let us now return to M. Guy de Maupassant. He was a man of genius, undoubtedly. But he has not had the sanity to preserve himself from insanity. On the contrary, if he had been determined of set purpose to become hopelessly mad, he could not have chosen a more speedy and effectual way of effecting his purpose than that which he pursued. M. de Maupassant is but forty-one years of age. A dozen years ago he was not only unknown to fame, but unknown to literature. Within the last ten years he has produced at least ten independent works of considerable pretensions. We are not here considering his works from a literary standpoint, but himself from a physiological point of view. He has been a literary cutting instrument with a fine edge, and therefore he demanded to be handled with great discretion and care lest his fine edge should be blunted and destroyed. But who was to handle him? He could only handle himself. When a man has reached the age of thirty he is no longer under tutors and governors; he fares forth on his own peculiar life journey under his own sole guidance and control. His mind may be of the most vivid brilliance, his objects may be of the noblest, but it is clear that a brilliant mind and noble purposes require to be guided and steadied by what, for want of a better term goes by the homely name of "saving common sense."

M. de Maupassant produced, as we have said, ten considerable independent works in ten years. To do that he must have worked his brain at high pressure. If he had taken the trouble to make himself master of a little of the elementary science of the times he would have known that ten years of brain work such as his demanded for the preservation of his thinking instrument certain positive counteractions. He should, for example, have limited himself strictly to a certain number of hours of daily work; he should have worked in the early part of the day in order that the abnormally active brain might be quieted down and rested before the arrival of the time for sleep. He should have taken a considerable amount of physical exercise every day for the purpose of balancing brain activity and blood-flow by muscular activity and blood-flow; he should have lived much out of doors in a pure atmosphere, so that the blood might have been well oxydised, the waste tissues clean carted off, and the brain and body highly nourished and thoroughly recuperated.

Instead of all this, what did M. de Maupassant do? He worked at his utmost capacity of speed and intensity. When he found himself flagging, he whipped his brain with stimulants instead of resting it. When he wanted rest he found that rest would not come; he could not rest naturally, and so he drugged himself into artificial rest, which was not rest, but the stupor of poison. All the papers speak as if M. de Maupassant had gone mad for the first time on the day when he attempted suicide. The physiologist, who knows best in these cases, considers that he went mad when he first began to whip himself to work with stimulants, and to drug himself to sleep with narcotics.

There is a terrible prospect before us all if we are to understand that any large proportion of writers are doing as M. de Maupassant has done. The power of journalists, novelists, and other writers, over the minds of all reading people is so profound and so vast as to be immeasurable. What if a majority of these are now doing as de Maupassant has been doing for the last few years, and are at the present time as truly insane as he has long been? They cannot keep their insanity out of their books and newspaper articles; it will mix with everything they write, like a subtle but most potent poison—a poison which comes from a judgment which is no longer sound, and which eats away, like a keen corrosive, other judgments with which it comes in contact: or a poison which is distilled from a moral faculty that has been ruined, and that inevitably tends to permeate and destroy other moral faculties which may try conclusions with it.

This is the prospect which the world has to face if its writers are not living, physically and mentally, wholesome and vigorous lives. But what of the prospect which the writers themselves have to face? They may see it in the story of M. Guy de Maupassant. He would have been a suicide if he could; he could not be a suicide, but he could be, and now is, a madman. The story is like a new star that has risen upon the literary horizon; a star that lights up a dark and dangerous prospect. Let every writer, who has eyes to see, open them and look.

